# Accidental lead in contaminated pipe-borne water and dietary furan intake perturbs rats’ hepatorenal function altering oxidative, inflammatory, and apoptotic balance

**DOI:** 10.1186/s40360-022-00615-0

**Published:** 2022-10-01

**Authors:** Solomon E. Owumi, Uche O. Arunsi, Omolola M. Oyewumi, Ahmad Altayyar

**Affiliations:** 1 ChangeLab-Changing Life Cancer Research and Molecular Biology Laboratories, Department of Biochemistry, Room NB302 Faculty of Basic Medical Sciences, University of Ibadan, Ibadan, Oyo 200004 Nigeria; 2 Department of Cancer Immunology and Biotechnology, School of Medicine, University of Nottingham, Nottingham, NG7 2RD UK

**Keywords:** Hepatorenal damage, Furan, Lead (II) acetate, Oxidative stress, Inflammation, Apoptosis

## Abstract

Inadvertent exposure to furan and Pb is associated with hepatorenal abnormalities in humans and animals. It is perceived that these two chemical species may work in synergy to orchestrate liver and kidney damage. Against this background, we investigated the combined effect of furan and incremental lead (Pb) exposure on hepatorenal dysfunction. Wistar rats (*n* = 30; 150 g) were treated for 28 days accordingly: Control; FUR (8 mg/kg), PbAc (100 µg/L), FUR + PbAc_1_ (8 mg/kg FUR + 1 µg/L PbAc); FUR + PbAc_1_ (8 mg/kg FUR + 10 µg/L PbAc), and FUR + PbAc_1_ (8 mg/kg FUR + 100 µg/L PbAc). Biomarkers of hepatorenal function, oxidative stress, inflammation, DNA damage, and apoptosis were examined. Furan and incrementally Pb exposure increased the levels of hepatorenal biomarkers and oxidative and pro-inflammatory mediators, including lipid peroxidation, reactive oxygen and nitrogen species, and interleukin-1 beta. Increased DNA damage, caspases- 9 and -3, and atypical histoarchitecture of the hepatorenal tissues exemplified furan and Pb treatment-related perturbations. Furthermore, the levels of antioxidants and IL-10 were also suppressed. Furan and Pb dose-dependently exacerbated hepatorenal derangements by altering the redox and inflammatory rheostats, worsened DNA damage, and related apoptotic onset that may potentiate hepatorenal disorders in humans and animals. The findings validate the synergistic effect of furan and Pb in the pathophysiology of kidney and liver disorders.

## Practical application

Failed public service and regulatory institutions in several countries worsen the sub-optimal public health oversight necessities. To date, corroded Pb-water pipes transport water to many homes, while food safety and drug regulatory agencies function nominally. Exposure ramifications exist in under-developed and developed countries, where highly processed furan-contamination food is consumed. We examined the inadvertent, albeit innocuous chronic exposure to furan from dietary sources and Pb contaminated water. Overall, exposure to furan and Pb adversely impacted rats’ hepatorenal functionality and integrity. Also, Furan and Pb exacerbated DNA damage, oxidative stress, and inflammatory responses, known to drive disease pathophysiology. The findings x-ray the danger of accidental exposure of Pb and furan to humans and animals. Based on this, we recommend that policy maker take heed and arrest an already untenable situation from becoming more problems that can become synonymous with the arsenic poisoning in Bangladesh a couple of decades ago. This combination of innocuously toxic exposure may be significant in the overall health condition of a susceptible population.

## Introduction

Anthropogenic activities are associated with the release of toxic chemicals into the environment. Some of these chemicals are easily biodegraded into less harmful metabolites and removed from the body through bile and urine. However, recalcitrant chemicals may accumulate in the body of animals and humans and orchestrate local and systemic toxicities over time [1–5]. In developing countries with no effective policies against the indiscriminate release of recalcitrant chemicals into the ecosystem, phytoplanktons, zooplanktons, and mammals are prone to heavy metal toxicities [2, 6, 7]. In recent years, thermal processing of food products has been observed to affect the structure and integrity of bioorganic molecules such as lipids, protein, carbohydrates, and nucleic acids present in foods, forming unpleasant compounds such as furan. Some literature pointed out that the thermal processing of foods could improve their antioxidant activity and total phenolic content [8]. However, recent observations revealed that elevated levels of degradative products such as furan in the body of animals and humans could potentiate systemic toxicities if not controlled [9–12].

Heavy metal such as lead (Pb) enters the food chain through anthropogenic activities such as gasoline mining, ore processing, lead-battery recycling, pipeline vandalisation, electric wastes, and vehicle exhausts [13, 14]. In the environment, Pb accumulates in phytoplankton [15]. When zooplanktons and mammals ingest phytoplankton, Pb bioavailability and bioaccumulation tend to increase systemically and could orchestrate severe toxicities if not prevented. Pb toxicities could be acute or chronic. During acute toxicity, Pb bioaccumulates in the bloodstream and is distributed into the liver, kidney, brain, and deep tissues, including the bones and cartilages in chronic toxicity [12, 16, 17]. From a toxicologist's perspective, there are no permissible limits for Pb. At low or high concentrations, this heavy metal is known to trigger systemic or local toxicities [11, 18] through the inhibition of phase-I and phase-II enzyme activities [19], mediation of oxidative and nitrosative stress and inflammation [20–23].

Furan is formed during the Maillard reaction, a non-enzymatic reaction of amino acids, peptides, and proteins with reducing sugars and vitamin C [24]. It is also a byproduct of the thermal degradation of carbohydrates, unsaturated fatty acids, amino acids, ascorbic acid, and carotenoids found in various foods such as coffee and canned and jarred foods [24–26]. Although Furan was regarded as a harmless food metabolite, recent assessments by the European Food Safety Authority (EFSA) correlated a high level of Furan to some human health abnormalities [27]. The knowledge of furan as a potent toxicant was further reinforced by the documentaries from the US Food and Drug Administration (US FDA), EFSA, National Toxicology Program (NTP), and International Agency for Research on Cancer (IARC). These Agencies highlighted evidence that exposure to Furan could orchestrate carcinogenesis in humans [24]. Furan toxicity is directly related to cytochrome P450 2E1 (CYP2E1) activities. ADMETically, Furan is acted upon by CYP2E1 into cis-but-ene-1,4-dialdehyde-a very reactive intermediate that interacts with amino acids, proteins, and DNA to induce toxicities in the liver and kidney [12]. In under-developed countries with no stringent regulations against heavy metal contamination and the degree of thermal processing of foods, Pb and Furan can be unintentionally ingested by humans and farm animals. Such accidental ingestion of Pb and furan could predispose victims to local and systemic toxicities. Based on this hypothesis, we put forward the following research question: can unfettered exposure of experimental rats to Pb and furan trigger oxidative, inflammatory, and apoptotic responses in the hepatorenal system, leading to their derangements? To answer these questions, adult male, Wistar Albino rats were exposed to lead acetate (PbAc) and furan for 28 d. Subsequently, the biomarkers of hepatorenal damage, oxidative stress, inflammation and apoptotic, oxidative DNA damage, and histological structural alteration in rats’ hepatic and renal tissues were evaluated.

## Materials and methods

### Chemicals, reagents and kits

The chemicals, reagents and kits used to determine the hepatorenal, redox, inflammatory and apoptotic biomarkers in the liver and kidney of rats following subacute exposure of rats to PbAc and Furan were purchased from recognised chemical companies. Specifically, Lead Acetate, Furan, TBA: thiobarbituric acid, DCFH-DA: 2’, 7’-dichlorodihydrofluorescin diacetate, DTNB: 5’, 5’-dithiobis-2-nitrobenzoic acid, CDNB: 1-chloro-2,4-dinitrobenzene, H_2_O_2_: hydrogen peroxide, KCl: potassium chloride, TCA: trichloroacetic acid, sodium azide, GSH: glutathione, epinephrine, sulphosalicylic acid, xanthine, Griess reagent, and O-dianisidine were purchased from Sigma-Aldrich Chemical (MO, USA); ALT: Alanine aminotransferase, AST: Aspartate aminotransferase, ALP: Alkaline phosphatase, urea, and creatinine were bought from Randox™ Laboratories Limited, (Crumlin, UK); and IL-1β: Interleukin 1-beta, IL-10: interleukin-10, 8-OHdG: 8-hydroxydeoxyguanosine, caspase-9 and caspase-3 were bought from Elabscience Biotechnology Company (Wuhan, China).

### Animal care, sample size, and experimental design

**Fig. 1:**
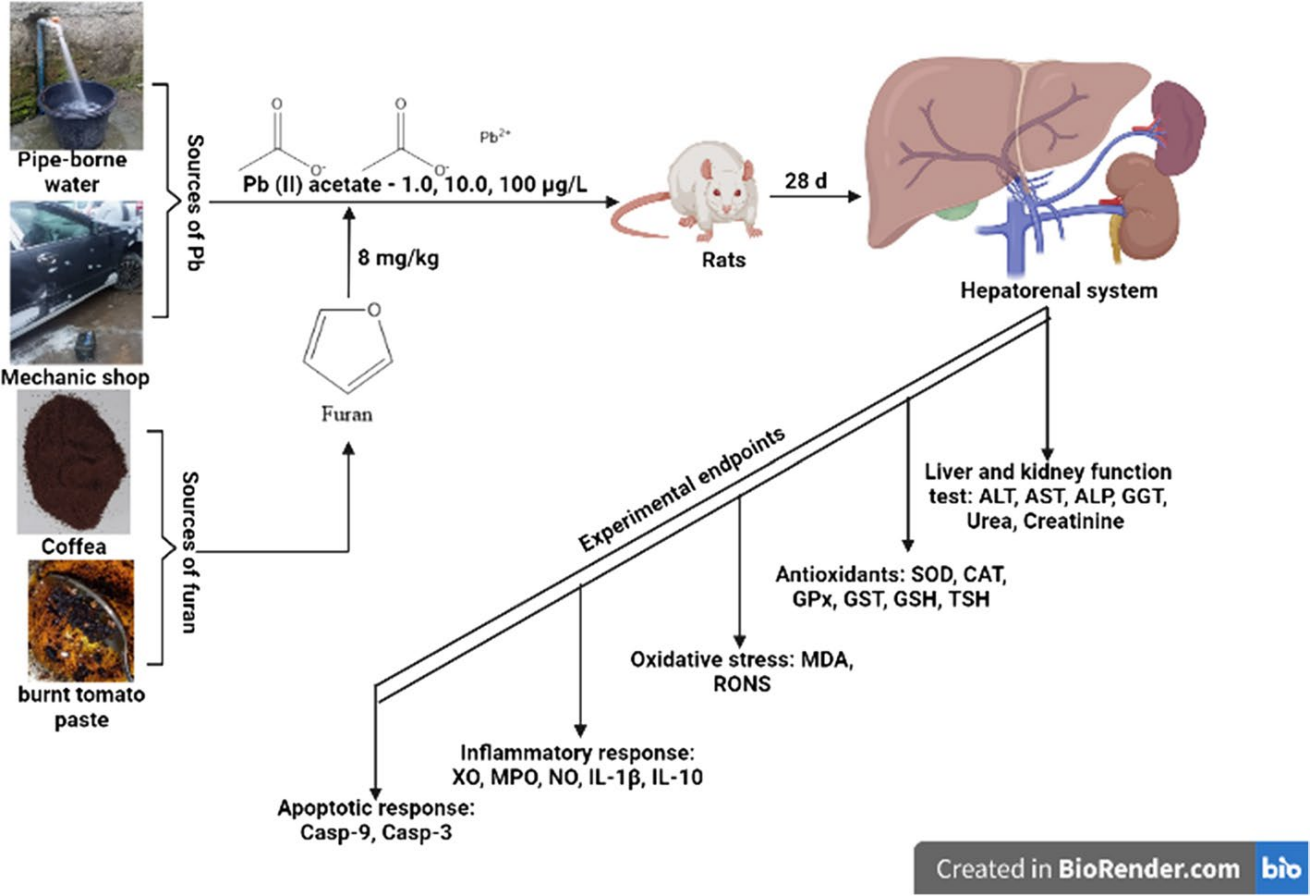
Experimental protocol of Lead and Furan-induced hepatorenal toxicities in rats for 28 consecutive days

The current study was conducted according to animal use guidelines in Life Sciences. The standard protocols for animal care and welfare (The 3Rs- replacement, reduction, and refinement) were adopted in this study as previously reported by Owumi et al. [28]. G* Power software version 3.1.9.4 [29] was used to estimate the sample size of 125 at an effect size of 0.40 and a 0.05 alpha error of probability for one-way analysis of variance (ANOVA). Out of 125 estimated experimental animals, 30 (consisting of *n* = 5 rats, i = 6) male Wistar Albino rats were sampled and used for the study. Rats weighing approximately 177 g per body weight (b.w.) were bought from the animal farms of the Faculty of Veterinary Medicine, University of Ibadan, Nigeria. The rats were carefully transferred to the animal house of the Department of Biochemistry, Faculty of Basic Medical Sciences, University of Ibadan, Nigeria, and maintained under a natural photoperiod of a daily 12 h darkness/light cycle. Experimental rats were fed with rat chows and clean water ad libitum and allowed to acclimate for 7 d before the execution of the experimental treatments. Rats were randomised into six experimental groups and subjected to 28 d successive treatments. Stock solutions for dosing experimental rats, Furan (8 mg/kg) and PbAc (0.1 mg/mL) were prepared daily. The doses of Furan and PbAc utilised in the present study were established from previously available data [6, 11]. Briefly, furan stock solution (8 mg/kg) was prepared by dissolving 213μL Furan in corn oil to make a total volume of 20 mL and then administered to rats *per os* (*po*) according to their body weight (with an average volume of 0.6 mL). PbAc stock solution (0.1 mg/ml) was prepared by dissolving 50 mg PbAc in 50 mL of distilled water. From this stock, 1.0, 10 and 100 μg/L were prepared by adding 0.02, 0.2 and 2 mL of PbAc and making up to 2L with distilled water. The volume of water was refilled daily to ensure a daily volume of 300 mL, and daily water intake was estimated after that. Rats in the group designated as the control received corn oil, Furan alone received 8 mg/kg body weight (b.w.) of Furan *p.o*, PbAc alone received 100 μg/kg bw of PbAc *p.o.,* Furan + PbAc_1_ received 8 mg/kg bw furan and 1 μg/kg b.w. PbAc, Furan + PbAc_2_ received 8 mg/kg b.w. furan and 10 μg/kg b.w. PbAc, and Furan + PbAc_3_ received 8 mg/kg b.w. furan and 100 μg/kg b.w. PbAc (Fig. 1). The study was carried out following the approval of the study proposal by the University of Ibadan Animal Care and Use Research Ethics Committee (ACUREC), with approval number UI-ACUREC/032–0525/27.

### Organ harvest, tissues processing, and experimental endpoints

At the end of the experiment, rats were starved for 24 h before animal sacrifice and experimental tissue and organ collection. Whole blood was collected from rats into plane tubes via retro-orbital venous plexus, and the rats were sacrificed by cervical displacement [30, 31]. Upon collection, whole blood was allowed to clot and centrifuged for 10 min at 3,000 rpm to obtain a clear serum needed to estimate the biomarkers of liver and kidney function tests. The liver and kidney were excised, weighed (USS-DBS16 Analytical Balance, Cleveland, OH, USA), and processed for biochemical and histological analyses. The relative organ weights of the liver and kidney were calculated according to the formula:

$$\mathrm{Relative\,organ \,weight }=\frac{\mathrm{Weight \,of \,organ }\left(\mathrm{g}\right)}{\mathrm{Weight \,of \,the \,body }\left(\mathrm{g}\right)} \mathrm{ x }100$$

Portions of the liver and kidney were used for biochemical and histological investigations. Samples for biochemical estimations were prepared by homogenising in a phosphate buffer (0.1 M, pH 7.4). The liver homogenate was prepared by homogenising 2 g of the liver in 8 mL of phosphate buffer, while the kidney homogenate was prepared by homogenising 1.12 g left or right kidney in 4 mL of phosphate buffer using a glass-Teflon homogeniser. The resultant homogenates were centrifuged at 12,000 rpm at 4^0^C for 15 min to get a clear mitochondrial fraction. The supernatants were collected in aliquots and frozen before quantifying oxidative, inflammatory, and apoptotic biomarkers.

### Determination of biomarkers of hepatorenal function biomarkers and of oxidative stress, inflammation, and DNA perturbation

The homogenate from the liver and kidney of the control, PbAc, and Furan-treated rats were subjected to biochemical analyses. The total protein concentrations of the liver and kidney were determined following the method of Bradford [32]. The liver and kidney function enzymes, including ALT, AST, ALP, creatinine and urea, were measured using commercial kits as previously reported by Owumi et al. [33]. Hepatic and renal concentrations of superoxide dismutase (SOD) were assayed by the methods described by Misra and Fridovich [34]; hepatic and renal levels of catalase (CAT) were assessed by the protocols of Clairborne [35] using H_2_O_2_ as a substrate; hepatic and renal levels of Glutathione-S-transferase (GST) and glutathione peroxidase (GPx) were measured by the procedures of Habig [36] and Rotruck et al., [37], respectively; hepatic and renal concentrations of glutathione (GSH) and total sulfhydryl group (TSH) were determined by the method of Jollow et al. [38] and Ellman [39], hepatic and renal concentrations of xanthine oxidase (XO) were measured by the procedures of Bergmeyer et al. [40]; hepatic and renal levels of malondialdehyde (MDA), otherwise termed lipid peroxidation (LPO) were assessed by the method described by Okhawa [41]; hepatic and renal levels of reactive oxygen and nitrogen species (RONS) were assayed by the protocols of Owumi and Dim [42]; hepatic and renal nitric oxide (NO) level and myeloperoxidase (MPO) activity were quantified by the protocols of Green et al. [43] and Granell et al. [44, 45], respectively; and hepatic and renal levels of IL-1β, IL-10, caspase-9 & -3 activities and 8-hydroxydeoxyguanosine were assayed using ELISA kits as previously reported by Owumi et al*.* [46]. All measurements were carried out using a Spectra Max™ plate reader.

### Examination of the histological sections of the liver and kidney

The portions of the liver and kidney kept for histological assessment were fixed in neutral buffered formalin (10%) before the histological section and staining preparation. With the aid of the standard paraffin-wax method, the liver and kidney tissues were processed for histopathological examination in line with the description of Bancroft and Gamble [47]. Approximately 5 μm thickness of the portion of the liver and kidney were dyed with haematoxylin and eosin and processed for light microscopy. All prepared slides were coded and probed with a Carl Zeiss Axio light microscope (Gottingen, Germany). On inspection, images were taken using a Zeiss Axiocam 512 camera (Gottingen, Germany) attached to the microscope by a pathologist unaware of the various treatment cohorts from which the slides were prepared.

### Statistical analysis of results

At the end of the experiments, data were generated, quantified, and subjected to statistical analyses using quantitative measures such as mean and standard deviation. The results were expressed as the mean ± SD of replicates. A test of statistical inferences was performed by student t-test to compare the significance between the IBW and FBW of rats, and a one-way analysis of variance (ANOVA) followed by a post-hoc test (Tukey’s test) set at a 95% probability level was used to test the significance difference across the four experimental groups using GraphPad Prism, version 8.3.0 for Mac (www.graphpad.com; GraphPad, CA, USA).

## Results

### Effects of combined exposure of Pb and Furan on rat’s body weight indices and hepatorenal biomarkers

**Fig. 2:**
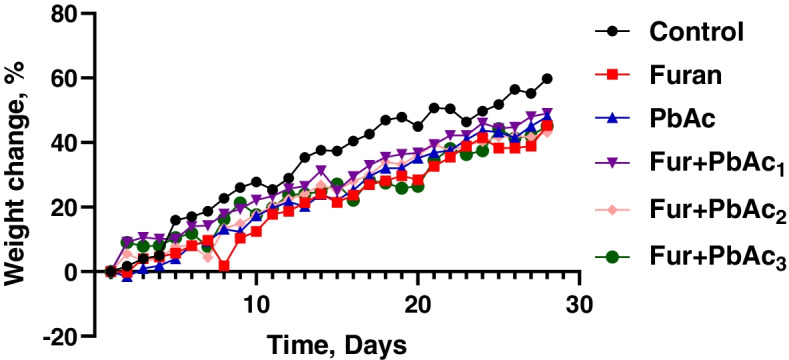
Effect of inadvertent co-exposure of experimental rats’ models to Lead and Furan in diet and water, respectively, on percentage weight gain

**Fig. 3:**
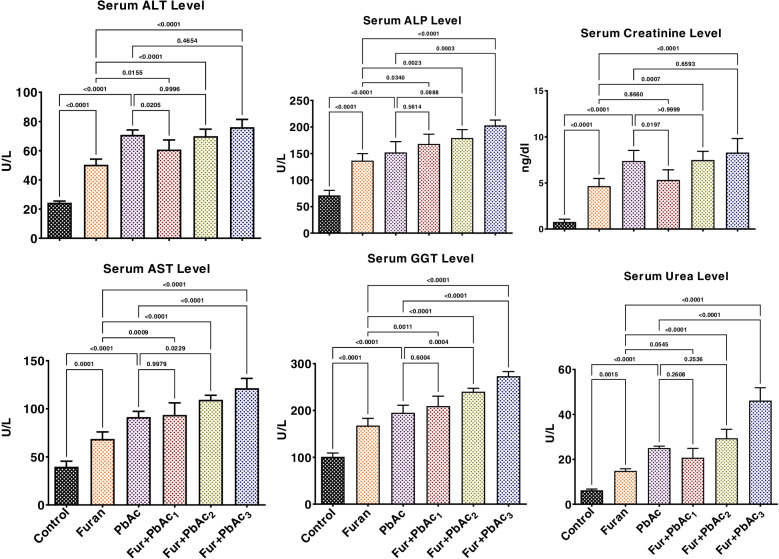
Effect of accidental co-exposure of experimental rats’ models to Lead and Furan in diet and water, respectively, on the activities of hepatorenal functional biomarkers in rats. Furan, 8 mg/kg; Lead, 100 μg/; Furan + PbAc_1_, (8 mg/kg + 1.0 μg/L) mg/kg; Furan + PbAc2, (8 mg/kg + 10 μg/L) mg/kg; Furan + PbAc_3_, (8 mg/kg + 100 μg/L) mg/kg. Values are expressed as mean ± SD for five rats per treatment cohort. The connecting lines indicate groups compared to one another, and the significance level was set at (*p* < 0.05); *p* < 0.05: indicates the level of significance; *p* > 0.05: Not significant. ALT: Alanine aminotransferase, AST: Aspartate aminotransferase: ALP: Alkaline phosphatase, GGT: Gamma-glutamyl transferase

The effects of Pb and Furan on rats’ body weight indices and hepatorenal biomarkers were examined in this study, and the results are presented in Table 1 and Figs. 1B and 2. In all cohorts of rats, the final body weights were significantly increased compared to the initial body weight. However, the mean body weight for cohorts of rats treated with PbAc and Furan decreased non-significantly compared to the control in the order Furan + PbAc_3_ < Furan + PbAc_2_ < Furan + PbAc_1_ < PbAc < furan < control (Fig. 2). Furthermore, the liver and kidney’s mean and relative organ weights were slightly altered following treatment in cohorts of rats treated with Pb and Furan compared to the control (Table 1). Biomarkers of hepatic toxicity -ALT, ALP, AST, and GGT; and kidney dysfunction -creatinine and urea- were significantly increased in rat cohorts treated with PbAc and Furan compared to the control (*p* < 0.0001). As the concentration of PbAc was raised, the activities of these enzymes were significantly elevated: ALT (*p* = 0.0205 in Furan + PbAc_1_ cohort), ALP (*p* = 0.0003 in Furan + PbAc_3_ cohort), AST (*p* = 0.0229 and *p* < 0.0001 in Furan + PbAc_2_ and Furan + PbAc_3_ cohorts, respectively), GGT (*p* = 0.0004 and *p* < 0.0001 in Furan + PbAc_2_ and Furan + PbAc_3_ cohorts, respectively), creatinine (*p* = 0.0197 in Furan + PbAc_1_ cohort, respectively), and urea (*p* < 0.0001 in Furan + PbAc_3_ cohort) (Fig. 3).

**Table 1:** Effect of the co-administration of furan and lead acetate on the body weight, organ weight and relative organ weight of experimental rats

	**Control**	**Furan**	**PbAc**	**F**ur + **PbAc**_1_	**F**ur + **PbAc**_**2**_	**Fur + PbAc**_**3**_
**IBW (g)**	152.60 ± 11.24	189.00 ± 10.34	193.60 ± 11.15	180.40 ± 9.71	169.80 ± 4.15	174.60 ± 3.58
**FBW (g)**	231.75 ± 18.80^#^	257.00 ± 7.00^#^	265.80 ± 8.53^#^	249.40 ± 15.47^#^	229.00 ± 14.78^#^	233.50 ± 12.50^#^
**Weight gain (g)**	79.20 ± 12.50	71.00 ± 6.60	69.20 ± 5.50	69.00 ± 7.91	59.20 ± 16.18	57.40 ± 14.05
**Liver weight (g)**	6.84 ± 1.00	7.72 ± 1.24^*^	7.68 ± 0.64	8.38 ± 0.77	8.72 ± 1.35	7.40 ± 0.93
**Kidney Weight (g)**	1.38 ± 0.15	1.40 ± 0.21^*^	1.60 ± 0.25	1.60 ± 0.13	1.44 ± 0.17	1.56 ± 0.22
**RLW (%)**	3.04 ± 0.19	3.73 ± 0.54	2.89 ± 0.21	3.36 ± 0.14	3.80 ± 0.51	3.21 ± 0.53
**RKW (%)**	0.62 ± 0.06	0.54 ± 0.09	0.60 ± 0.09	0.64 ± 0.02	0.63 ± 0.10	0.67 ± 0.09

FUR (8 mg/kg), PbAc (100 µg/kg b.w.), PbAc_1_ (1 µg/kg b.w.), PbAc_2_ (10 µg/kg b.w.), PbAc_3_ (100 µg/kg b.w.); n = 5. Data are expressed as mean ± SD

*Fur* Furan, *IBW* Initial body weight, *FBW* Final Body weight, *RLW* Relative liver weight, *RKW* Relative kidney weight

(IBW versus FBW: ^#^*p* < 0.05)

(Control versus Furan or PbAc: ^*^p < 0.05)

### Effects of combined treatment of Pb and Furan on redox balance in the liver and kidney of rats

**Fig. 4:**
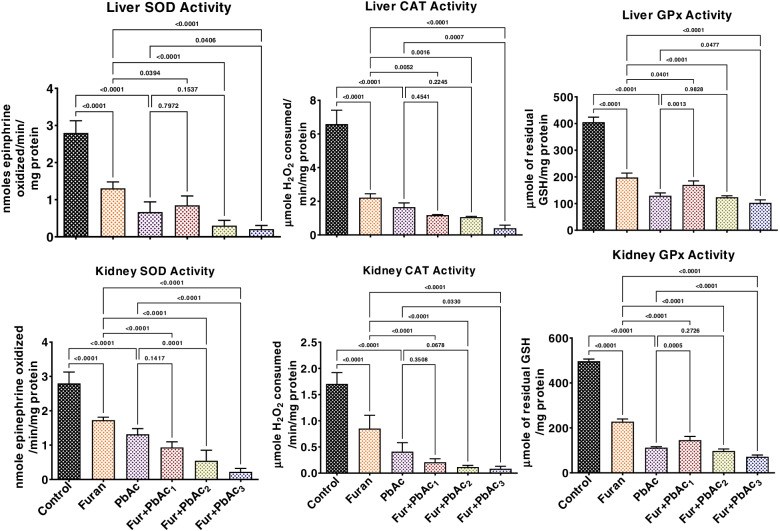
Effect of accidental co-exposure of experimental rats’ models to Lead and Furan in diet and water respectively on the hepatic and renal activities of SOD, CAT and GPx in rats. Furan, 8 mg/kg; Lead, 100 μg/; Furan + PbAc_1_, (8 mg/kg + 1.0 μg/L) mg/kg; Furan + PbAc2, (8 mg/kg + 10 μg/L) mg/kg; Furan + PbAc_3_, (8 mg/kg + 100 μg/L) mg/kg. Values are expressed as mean ± SD for five rats per treatment cohort. The connecting lines indicate groups compared to one another; the significance level was set at (*p* < 0.05); *p* < 0.05: indicates the level of significance; *p* > 0.05: Not significant. SOD: Superoxide dismutase, CAT: Catalase, GPx: Glutathione peroxidase

**Fig. 5:**
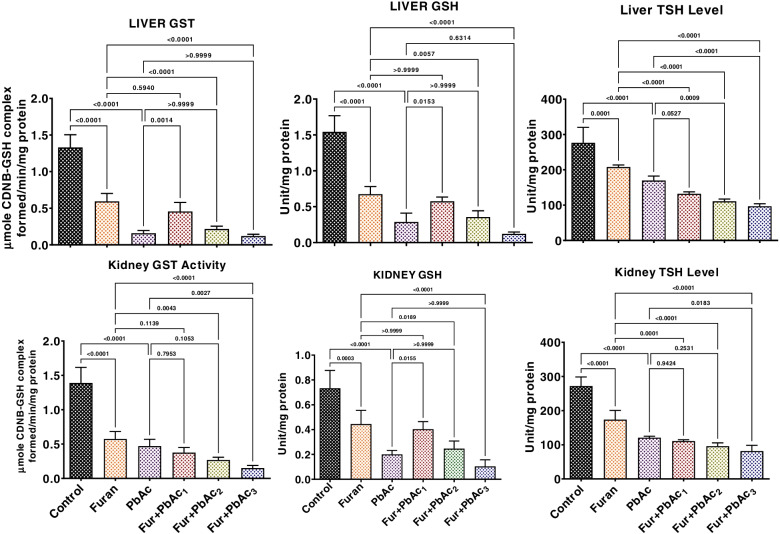
Effect of accidental co-exposure of experimental rats’ models to Lead and Furan in diet and water respectively on the hepatic and renal levels of GPx, GSH and TSH in rats. Furan, 8 mg/kg; Lead, 100 μg/; Furan + PbAc_1_, (8 mg/kg + 1.0 μg/L) mg/kg; Furan + PbAc2, (8 mg/kg + 10 μg/L) mg/kg; Furan + PbAc_3_, (8 mg/kg + 100 μg/L) mg/kg. Values are expressed as mean ± SD for five rats per treatment cohort. The connecting lines indicate groups compared to one another, and the significance level was set at (*p* < 0.05); *p* < 0.05: indicates the level of significance; *p* > 0.05: Not significant. GST: Glutathione S-transferase, GSH: reduced Glutathione, TSH: Total sulfhydryl group

**Fig. 6:**
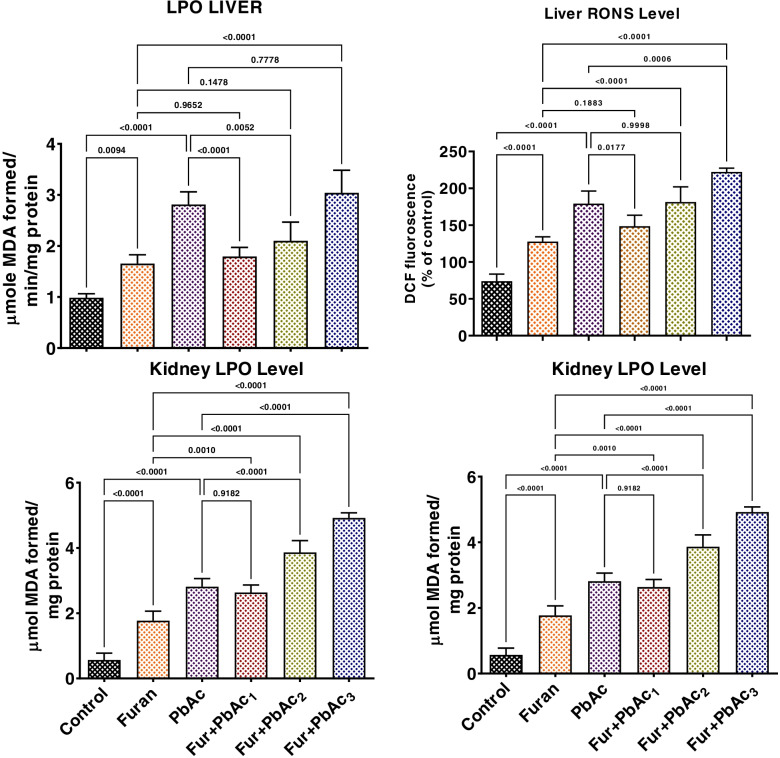
Effect of accidental co-exposure of experimental rats’ models to Lead and Furan in diet and water, respectively, on the hepatic and renal levels of LPO and RONS in rats. Furan, 8 mg/kg; Lead, 100 μg/; Furan + PbAc_1_, (8 mg/kg + 1.0 μg/L) mg/kg; Furan + PbAc2, (8 mg/kg + 10 μg/L) mg/kg; Furan + PbAc_3_, (8 mg/kg + 100 μg/L) mg/kg. Values are expressed as mean ± SD for five rats per treatment cohort. Connecting lines indicate groups compared to one another. The significance level was set at (*p* < 0.05); *p* < 0.05: indicates the level of significance; *p* > 0.05: Not significant. LPO: Lipid peroxidation, RONS: Reactive oxygen and nitrogen species

The effects of combined Pb and Furan treatment on antioxidant and oxidative biomarkers in rat liver and kidney were probed in this study, as presented in Figs. 3 and 4. Exposure to Pb and Furan separately to rats significantly waned the tissue concentrations of SOD, CAT, GPx, GST, GSH, and TSH in the liver and kidney of rats compared to the control (*p* < 0.0001) (Figs. 4 and 5). Interestingly, the decline the levels of these antioxidant biomarkers were further significantly enhanced when the concentrations of PbAc was raised, while maintaining the amount of furan in cohorts of rats treated with PbAc and furan: SOD (*p* = 0.0406 in the liver of Furan + PbAc_3_ treated cohort; *p* = 0.0001 and *p* < 0.0001 in the kidney of Furan + PbAc_2_ and Furan + PbAc_3_ treated cohorts, respectively), CAT (*p* = 0.0007 in the liver of Furan + PbAc_3_ treated cohort; *p* = 0.0001 in the kidney of Furan + PbAc_3_ treated cohort), GPx (*p* = 0.0013 and *p* = 0.0477 in the liver of Furan + PbAc_1_ and Furan + PbAc_3_ treated cohorts, respectively; *p* = 0.005 and *p* < 0.0001 in the kidney of Furan + PbAc_1_ and Furan + PbAc_3_ treated cohorts, respectively) (Fig. 4), GST (*p* = 0.0014 in the liver of Furan + PbAc_1_ treated cohort; *p* = 0.0027 in the kidney of Furan + PbAc_3_ treated cohort), GSH (*p* = 0.0153 in the liver of Furan + PbAc_1_ treated cohort; *p* = 0.0155 in the kidney of Furan + PbAc_1_ treated cohort), and TSH (*p* = 0.0009 and *p* < 0.0001 in the liver of Furan + PbAc_2_ and Furan + PbAc_3_ treated cohorts, respectively; *p* = 0.0183 in the kidney of Furan + PbAc_3_ treated cohort) (Fig. 5). The results further reveal that the exposure to PbAc and Furan significantly elevated the hepatic and renal levels of LPO and RONS (*p* < 0.0001) in a cohort of rats compared to the untreated group. However, as the concentration of PbAc was increased, the levels of LPO and RONS significantly elevated in cohorts of rats treated with PbAc and furan: LPO (*p* < 0.0001 and *p* = 0.0062 in the liver of Furan + PbAc_1_ and Furan + PbAc_2_ treated cohorts, respectively; *p* < 0.0001 in the kidney of Furan + PbAc_2_ and Furan + PbAc_3_ treated cohorts), and RONS (*p* = 0.0177 and *p* = 0.0005 in the liver of Furan + PbAc_1_ and Furan + PbAc_3_ treated cohorts, respectively; *p* < 0.0001 in the kidney of Furan + PbAc_2_ and Furan + PbAc_3_ treated cohorts) (Fig. 6).

### Effect of combined treatment of Pb and Furan on the inflammatory response, oxidative DNA damage and apoptosis in the liver and kidney of rats

**Fig. 7:**
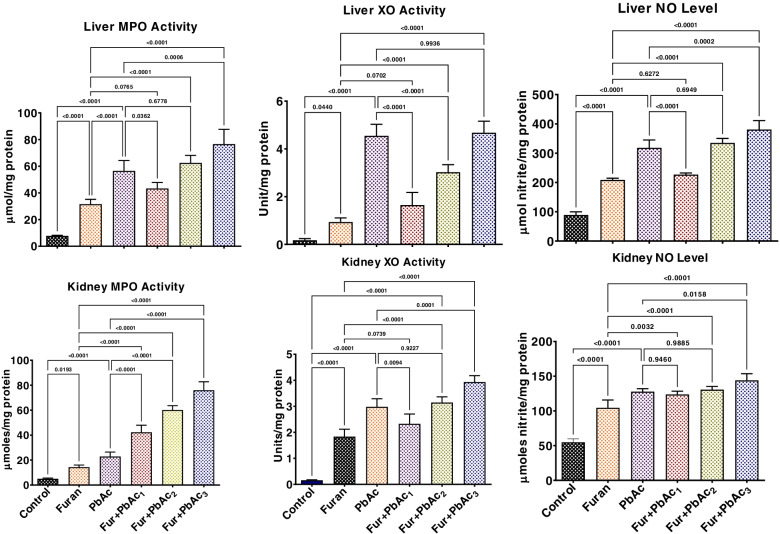
Effect of accidental co-exposure of experimental rats’ models to Lead and Furan in diet and water, respectively, on the hepatic and renal levels of XO, MPO, and NO in rats. Furan, 8 mg/kg; Lead, 100 μg/; Furan + PbAc_1_, (8 mg/kg + 1.0 μg/L) mg/kg; Furan + PbAc2, (8 mg/kg + 10 μg/L) mg/kg; Furan + PbAc_3_, (8 mg/kg + 100 μg/L) mg/kg. Values are expressed as mean ± SD for five rats per treatment cohort. The connecting lines indicate groups compared to one another, and the significance level was set at (*p* < 0.05); *p* < 0.05: indicates the level of significance; *p* > 0.05: Not significant. XO: xanthine oxidase, MPO: Myeloperoxidase, NO: Nitric oxide

**Fig. 8:**
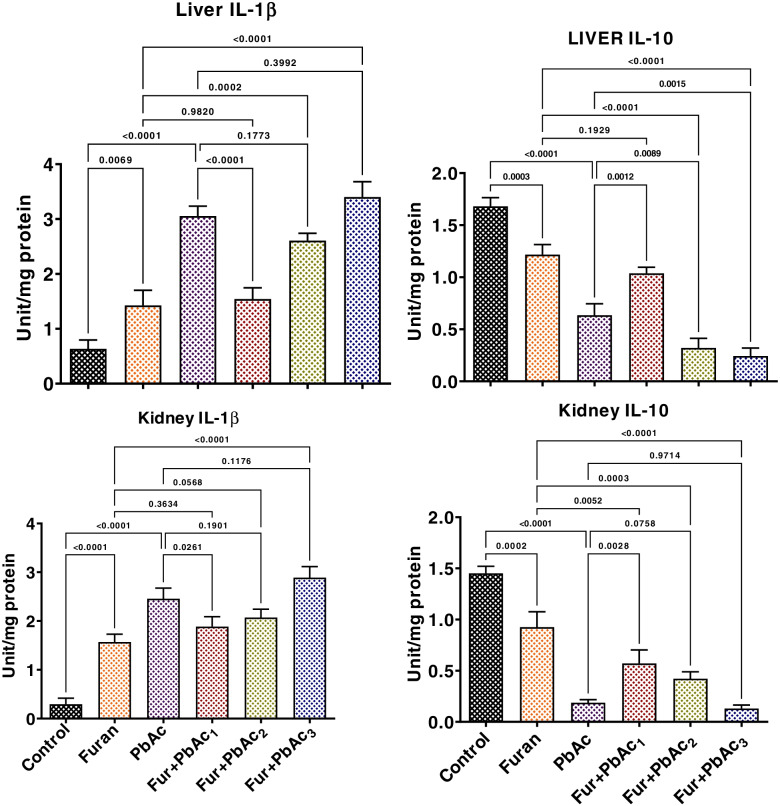
Effect of accidental co-exposure of experimental rats’ models to Lead and Furan in diet and water respectively on the hepatic and renal levels of IL-1β and IL-10 in rats. Furan, 8 mg/kg; Lead, 100 μg/; Furan + PbAc_1_, (8 mg/kg + 1.0 μg/L) mg/kg; Furan + PbAc2, (8 mg/kg + 10 μg/L) mg/kg; Furan + PbAc_3_, (8 mg/kg + 100 μg/L) mg/kg. Values are expressed as mean ± SD for five rats per treatment cohort. The connecting lines indicate groups compared to one another, and the significance level was set at (*p* < 0.05); *p* < 0.05: indicates the level of significance; *p* > 0.05: Not significant. IL-1β: Interleukin 1-beta, IL-10: Interleukin 10

**Fig. 9:**
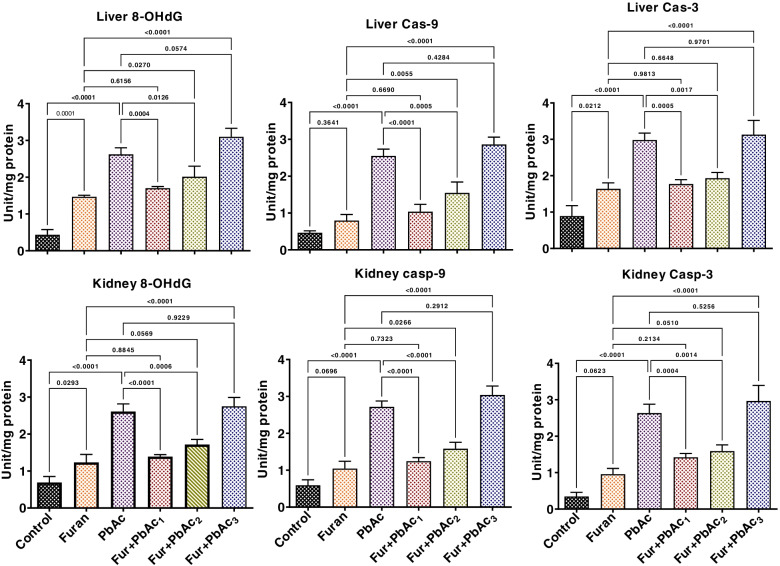
Effect of accidental co-exposure of experimental rats’ models to Lead and Furan in diet and water respectively on the hepatic and renal levels of 8-OHdG, Casp-9 and Casp-3 in rats. Furan, 8 mg/kg; Lead, 100 μg/; Furan + PbAc_1_, (8 mg/kg + 1.0 μg/L) mg/kg; Furan + PbAc2, (8 mg/kg + 10 μg/L) mg/kg; Furan + PbAc_3_, (8 mg/kg + 100 μg/L) mg/kg. Values are expressed as mean ± SD for five rats per treatment cohort. The connecting lines indicate groups compared to one another, and the significance level was set at (*p* < 0.05); *p* < 0.05: indicates the level of significance; *p* > 0.05: Not significant. 8-OHdG: 8-hydroxydeoxyguanosine, Casp: Caspase

The study also probed the abrogative roles of the combined treatment of Pb and Furan on rats’ inflammatory and apoptotic responses and oxidative DNA damage. The results are presented in Figs. 6 and 7. Administration of separate doses of PbAc and Furan to cohorts of rats significantly elevated the hepatic and renal levels of proinflammatory mediators such as MPO, XO, and NO (*p* < 0.0001). As the concentration of PbAc was raised at a constant concentration of furan, the hepatic and renal levels of these pro-inflammatory molecules were significantly elevated in cohorts of rats treated with PbAc and furan: MPO (*p* = 0.0362 and *p* = 0.0006 in the liver of Furan + PbAc_1_ and Furan + PbAc_2_ treated cohorts, respectively; *p* < 0.0001 in the kidney of Furan + PbAc_1,_ Furan + PbAc_2_, and Furan + PbAc_3_ treated cohorts), XO (*p* < 0.0001 in the liver of Furan + PbAc_1_ and Furan + PbAc_3_ treated cohorts; *p* = 0.0094 and *p* = 0.0001 in the kidney of Furan + PbAc_2_ and Furan + PbAc_3_ treated cohorts, respectively), and NO (*p* < 0.0001 and *p* = 0.0002 in the liver of Furan + PbAc_1_ and Furan + PbAc_3_ treated cohorts, respectively; *p* = 0.0158 in the kidney of Furan + PbAc_3_ treated cohort) (Fig. 7). Similarly, treatments with Pb and Furan alter the levels of pro-inflammatory and anti-inflammatory molecules in the liver and kidney of cohorts of rats. In contrast to the control, PbAc and furan significantly increased the hepatic and renal levels of IL-1β. Still, they decreased the hepatic and renal levels of IL-10, indicating an alteration in the basal inflammatory responses: IL-1β (*p* < 0.0001 in the liver of Furan + PbAc_1_ treated cohort; *p* = 0.0261 in the kidney of Furan + PbAc_1_ treated cohort) and IL-10 (*p* = 0.0012, *p* = 0.0089, and *p* = 0.0015 in the liver of Furan + PbAc_1,_ Furan + PbAc_2_ and Furan + PbAc_3_ treated cohorts, respectively; *p* = 0.0028 in the kidney of Furan + PbAc_1_ treated cohort) (Fig. 8). In experimental animals, increased oxidative and inflammatory responses can result in oxidative DNA damage and apoptosis. We further probed Pb and Furan’s effect on 8-OHdG and caspase-9 and caspase-3 levels, as presented in Fig. 9. Treatments with Pb and Furan significantly increased the hepatic and renal levels of 8-OHdG and the activities of caspase -9 and -3 in cohorts of rats treated with different doses of PbAc and Furan relative to the control. Intriguingly, as the dosage of PbAc raised at constant dosage of furan, the level of 8-OHdG and activities of caspase-9 and -3 in the liver and kidney of rats markedly increased, an indication of oxidative DNA damage and apoptosis: 8-OHdG (*p* = 0.0004, *p* = 0.0126, and *p* = 0.057 in the liver of Furan + PbAc_1,_ Furan + PbAc_2_ and Furan + PbAc_3_ treated cohorts respectively; *p* < 0.0001 and *p* = 0.0006 in the kidney of Furan + PbAc_1_ and Furan + PbAc_2_ treated cohorts, respectively), caspase-3 (*p* < 0.0001 and *p* = 0.0017 in the liver of Furan + PbAc_1_ and Furan + PbAc_2_ treated cohorts respectively; *p* < 0.0001 and *p* < 0.0001 in the kidney of Furan + PbAc_1_ and Furan + PbAc_2_ treated cohorts, respectively), and caspase-3 (*p* = 0.0005 and *p* = 0.0017 in the liver of Furan + PbAc_1_ and Furan + PbAc_2_ treated cohorts respectively; *p* = 0.0005 and *p* = 0.0014 in the kidney of Furan + PbAc_1_ and Furan + PbAc_2_ treated cohorts, respectively) (Fig. 9).

### Effect of combined treatment of Pb and Furan on histological features of rat’s liver and kidney

**Fig. 10:**
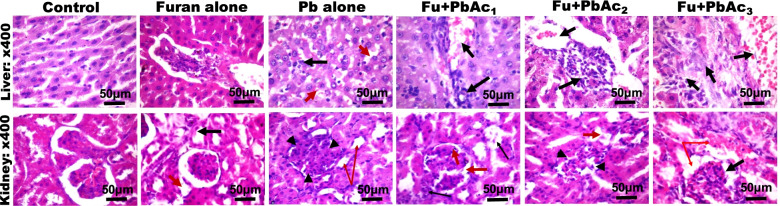
Photomicrograph of tissue _liver and kidney section stained with H and E and a magnification: × 400; depicting alterations in the tissue of rats exposed to environmentally relevant concentrations of lead (PbAC3) and furan for 28 consecutive days. Column 1: Control: Liver and kidney tissue histoarchitecture were typical without unusual characteristics. The liver sinusoids appear normal and not infiltrated. However, there is a significant presence of inflammatory cells in the kidney. Column 2: Furan alone: Liver and kidney sections appear normal, with the renal cortex showing normal glomeruli, mesangial cells and capsular spaces (red arrow). Also, the renal tubules appear normal (black arrow), and the interstitial spaces appear normal (slender arrow). Column 3: Lead alone: Liver: the morphology of the hepatocytes at zone 3 shows spongy and balloon-like cytoplasm (red arrow), while other liver cells appear normal (black arrow). The hepatic sinusoids appear normal and without infiltration of any cell type. The kidney showed poor architecture at a lower magnification (× 100) image not shown, and the renal cortex showed normal glomeruli with normal mesangial cells and capsular spaces (black arrowheads) with collapsed renal tubules (red arrow). The interstitial spaces show the focal area of accumulated eosinophilic fluid (slender red arrow). Column 4–6: Furan and Pb with varying doses (1–3): Liver histoarchitecture of Pb1-3: progressive congestion around the portal vein (black arrow) was observed in the liver of the experimental rats, while the morphology of the sinusoids appears normal without any significant infiltration by inflammatory cells. The kidney histoarchitecture remains intact, and the renal cortex shows normal glomeruli with normal mesangial cells and capsular spaces (black arrow). The renal tubules and the interstitial spaces appear normal (red arrow)

Finally, we investigated the effect of the combined treatment of Pb and Furan on the histological features of the liver and kidney of rats after 28 days (Fig. 10). Unlike the control cohort, which shows normal histoarchitectural features such as typical liver sinusoids, and renal tubules with basal levels of inflammatory cells, the liver and kidney of rats treated with Furan appear normal. The renal cortex showed normal glomeruli, mesangial cells and capsular spaces (red arrow). Also, the renal tubules (black arrow) and the interstitial spaces seemed normal (slender arrow). At the same time, the cohort of rats treated with PbAc alone manifested spongy and balloon-like cytoplasm (red arrow) in hepatic Zone 3, while other cells appeared normal (black arrow) in the liver. The kidney tissues with the renal cortex depict normal glomeruli, mesangial cells and capsular spaces (black arrowheads), and collapsed renal tubules (red arrow). The interstitial spaces show a focal area accumulated with eosinophilic fluid (slender red arrow). At higher doses of PbAc and a constant dose of Furan, the histoarchitectural features of the liver and kidney deviated from the normal features seen in the control cohort. Liver histoarchitecture of Furan at PbAc_1-3_ manifested progressive congestion around the portal vein (black arrow), although the morphology of the sinusoids appears normal without any significant infiltration by inflammatory cells. The kidney histoarchitecture remains intact, and the renal cortex shows normal glomeruli with normal mesangial cells and capsular spaces (black arrow). The renal tubules and the interstitial spaces appear normal (red arrow).

## Discussion

In this study, we investigated whether the exposure of rats to PbAc and Furan could perturb redox balance and orchestrate oxidative DNA damage and pro-inflammatory and apoptotic responses after 28 d. Our results reveal that exposure of the experimental rats to PbAc and Furan altered redox balance in the hepatorenal system. Loss of redox homeostasis resulted in oxidative DNA damage and triggered pro-inflammatory and apoptotic responses. The manifestation of hepatorenal derangements is validated by increased liver and kidney function biomarkers and loss of typical histoarchitectural features in rat liver and kidney. These findings support previous works that the administration of PbAc and Furan to experimental animals could upset the balance between antioxidants and pro-oxidants, trigger inflammation and apoptosis, and alter the basal level of hepatic and renal functional enzymes and typical histological features in the liver and kidney of rats [12, 48, 49].

Our observations that exposure to Pb and Furan is associated with hepatorenal toxicities indicate the existence of multiple mechanistic routes for Pb and furan-mediated toxicities, including induction of oxidative stress, oxidative DNA damage, chronic inflammation, and apoptosis. Oxidative stress (OS) occurs when there is a disparity between antioxidants and pro-oxidants in favour of pro-oxidant production [50]. OS is triggered following exposure to pathogens and toxic chemicals. OS assists pathogen clearance at the basal level and regulates essential biochemical and physiological processes. However, it becomes harmful to healthy cells and tissues when in excess, as observed in the current study. The OS state begins with the release of free radicals, especially superoxide ion radical (O2^.−^), during phase 1 of the biotransformation of xenobiotics [51, 52]. An increase in hepatic and renal levels of this radical triggers the expression of an endogenous antioxidant, SOD, which is known to mediate the dismutation of (O2^.−^) into a less toxic free radical—hydrogen peroxide (H_2_O_2_) [53]. Excess H_2_O_2_ in the hepatocytes and nephrons has been shown to trigger the expression of GPx and CAT, known to break down excess H_2_O_2_ into molecular water. However, repression in the expression of these innate antioxidant molecules can predispose the liver and kidney to severe toxicities [54, 55], as observed in the current study. When the expressions of endogenous antioxidant enzymes decrease, non-enzymatic antioxidants, including GSH and TSH, can scavenge the excess free radicals.

Consequently, switching the redox state towards a reducing condition helps scavenge reactive hydroxyl radicals, oxygen-centred radicals, and radical centres on DNA. GSH can either serve as a co-substrate of GPx and permit the reduction of peroxides (hydrogen and lipid peroxides) or conjugate with electrophilic endogenous compounds (xenobiotics and their phase I metabolites) in GST-mediated reaction for easy excretion [56, 57]. However, depletion in the hepatic and renal levels of GST indicates an influx in ROS in target tissues, leading to cellular and tissue toxicities, as seen in the current study. Due to reduced antioxidants, H_2_O_2_ may accumulate in the hepatocytes and nephrons, triggering the formation of hydroxyl radical (OH^−^) in the presence of iron or copper [58]. These radicals and many others can react with membrane lipids, proteins, and nucleic acids, triggering lipid peroxidation and disrupting cellular integrity. The current data shows that a high MDA (LPO) and other RONS with significant diminution in the antioxidant buffering system indicate hepatorenal derangement [46, 59].

The pro-inflammatory response is another route of Fe and Furan-mediated liver and kidney toxicities [60, 61]. Several mediators drive inflammation, including MPO, XO, NO, IL-1β, and IL-10. Significant alterations in hepatic and renal levels of these molecules may trigger the onset of inflammation, as observed in cohorts of rats treated with PbAc and Furan. MPO is a member of haem peroxidase expressed on neutrophils and monocytes [62] and is known to catalyse the conjugation of H_2_O_2_ and chloride, to yield hypochlorous acid (HOCl) [63]. On decomposition, HOCl evolves harmful free radicals such as singlet oxygen (^1^O_2_) and^**.**^OH [64]. These free radicals bind to biological molecules, including lipids, proteins, and nucleic acids in the hepatocytes and nephrons and form lipid peroxides, protein crosslinks and DNA adducts [65]. Excess breakdown of nucleic acid may release free purine bases, which activate the expression of XO. XO then catalyses the breakdown of hypoxanthine and xanthine with the evolution of urea acid and O2^−^. O2^−^ produced in this reaction sequence further provokes the hepatorenal system’s redox balance. At the same time, uric acid may serve as a buffer to render temporary antioxidant protection [66], as a dangerous signal to activate many inflammasome pathways or drive the expression of several inflammatory cytokines in the hepatocytes and nephrons of the experimental rats [67, 68]. Another marker of pro-inflammation is NO, depending on its cellular and tissue concentrations.

NO mediates essential biochemical and physiological effects at a normal concentration, such as vasodilation, neurotransmission, and immune system function [69]. However, in toxin-ridden cells and tissues, as observed in our study, alteration in redox balance triggers increased expression of nitric oxide synthase (NOS). This enzyme catalyses the conversion of L-arginine into L-ornithine and NO in the presence of reduced nicotinamide adenine dinucleotide phosphate (NADPH) and molecular O_2_ as co-factors [70]. An increase in NOS expression level will, in turn, drive the production of NO beyond its basal level. NO can promote the oxidative state in hepatic and renal systems by conjugating with O2^−^ to form peroxynitrite (ONOO^−^). An increase in the activities of these free radicals can trigger the activation of several innate cells of the immune system, thereby increasing the pro-inflammatory milieu needed to enhance hepatic and renal damage [46, 71], as seen in the present study. As the activities of these pro-inflammatory mediators increase, they trigger the generation of several danger signals, which interact with cells of the immune system resident in the hepatic and renal tissues, notably the Kupffer cells and intraglomerular mesangial cells, respectively. This interaction may activate NF-κB, which exits the cytoplasm and translocates into the nucleus. This reaction sequence will facilitate the transcription of cytokines genes, including IL-1β and other pro-inflammatory cytokines. IL-1β, in collaboration with TNF-α, IL-6 and IL-8, participate in the acute phase response and triggers severe hepatic and renal damage, as observed in the present study. As a resolvin, IL-10—an anti-inflammatory cytokine- suppresses pro-inflammatory responses [72]. Still, where the expression of IL-10 is dampened, as seen in this study, the hepatic and renal tissue is exposed to more inflammatory insults, leading to hepatorenal derangements [73]. The involvement of Pb and Furan in mediating inflammation and oxidative stress in rats' liver and kidneys have been previously studied in rats [12, 48, 49].

Furan and Pb-mediated oxidative stress and inflammation can trigger oxidative DNA damage, leading to the formation of DNA adducts such as 8-OHdG, as observed in the study. Increases in hepatorenal 8-OHdG levels can trigger the transcriptional activation of p53—the genome guardian. Activated p53 can stall the cell cycle and induce DNA repair pathways. Cell cycle arrest is necessary to remove damaged nucleotides or initiate programmed cell death when DNA repair is not feasible [74, 75]. Precisely, p53 activates PUMA, which activates Bax while inhibiting Bcl-2 [76]. Bax then translocates into the hepatocytes and nephrons’ mitochondria and triggers the release of cytochrome C, which binds to apoptotic peptidase activating factor 1 (APAF-1) and pro-caspase-9 to form apoptosome. This complex mediates the activation of caspase-9, the initiator of apoptosis, and caspase-9, in turn, activates caspase-3—the executioner of apoptosis. As this study reveals, increased caspase-9 and -3 activities imply programmed cell death in the liver and kidney of rats. These observations agree with the findings from previous studies that xenobiotics can mediate apoptosis following redox imbalance and increase pro-inflammatory mediators [46, 77, 78].

Unabated increase in the level of ROS, pro-inflammatory mediators, and caspases with concurrent wane in the expression of phase 1 and phase 11 antioxidants, and anti-inflammatory cytokine, the liver and kidney can predispose the liver to severe toxicities, including distortion of the hepatic and renal membrane lipids and proteins, and loss of function and integrity of proteins and nucleic acids in the liver and kidney of rats. These changes can result in hepatic and renal damage, as observed in the present study. One way to clinically elucidate that the liver and kidney are prone to disease following treatment with PbAc and Furan is by evaluating the serum concentrations of liver and kidney functional biomarkers such as ALT, AST, ALP, GGT, urea and creatinine. The levels of these molecules in the serum are stringently regulated. In a normal liver, ALT, AST, ALP and GGT are localised in specific cellular compartments, including the cytoplasm and mitochondria. However, these enzymes are released into the circulation in a disease state, indicating hepatic damage, as observed in the present study. In addition, creatinine, and urea – the biomarkers of renal function, are produced from creatinine phosphate and the breakdown of proteins, respectively. Following kidney injury, these metabolites accumulate in the bloodstream due to impaired excretion, and if this is not remedied, it can trigger renal failure. The implications of ALT, AST, ALP, GGT, urea, and creatinine in acute/chronic liver and kidney diseases have been reported previously [11, 48, 65, 73].

Another clinically accepted protocol for ascertaining xenobiotics' toxicities is evaluating a target organ's histoarchitectural features. In this study, treatments with PbAc and Furan abrogated the normal histology of the liver and kidney, and this may be attributed to the increase in the generation of ROS, proinflammatory cytokines, DNA adducts, and a host of other danger-associated molecular patterns (DAMPs) [46, 63, 73]. These molecules are known to trigger the activation and differentiation of pro-inflammatory cells in the hepatic and renal tissues, leading to the activation of inflammasomes [79, 80] and the formation of lesions capable of deranging the liver and kidney of rats. These parameters are relevant in clinical diagnosis. However, some non-invasive measures exist, such as body weight and organosomatic indices, for validating the impact of chemical toxicants on experimental models [81]. In this study, exposure to PbAc and Furan did not significantly alter the organosomatic indices of rats after 28 days. However, we infer that long-term exposure may trigger a decrease in the body weight of rats and the weight of the liver and kidneys. Based on these indices, we revalidate the health impact of Pb and furan exposure on animals and confirm that these xenobiotics could trigger oxidative stress, chronic inflammation, oxidative DNA damage, and apoptosis at the test doses.

## Conclusion

**Fig. 11:**
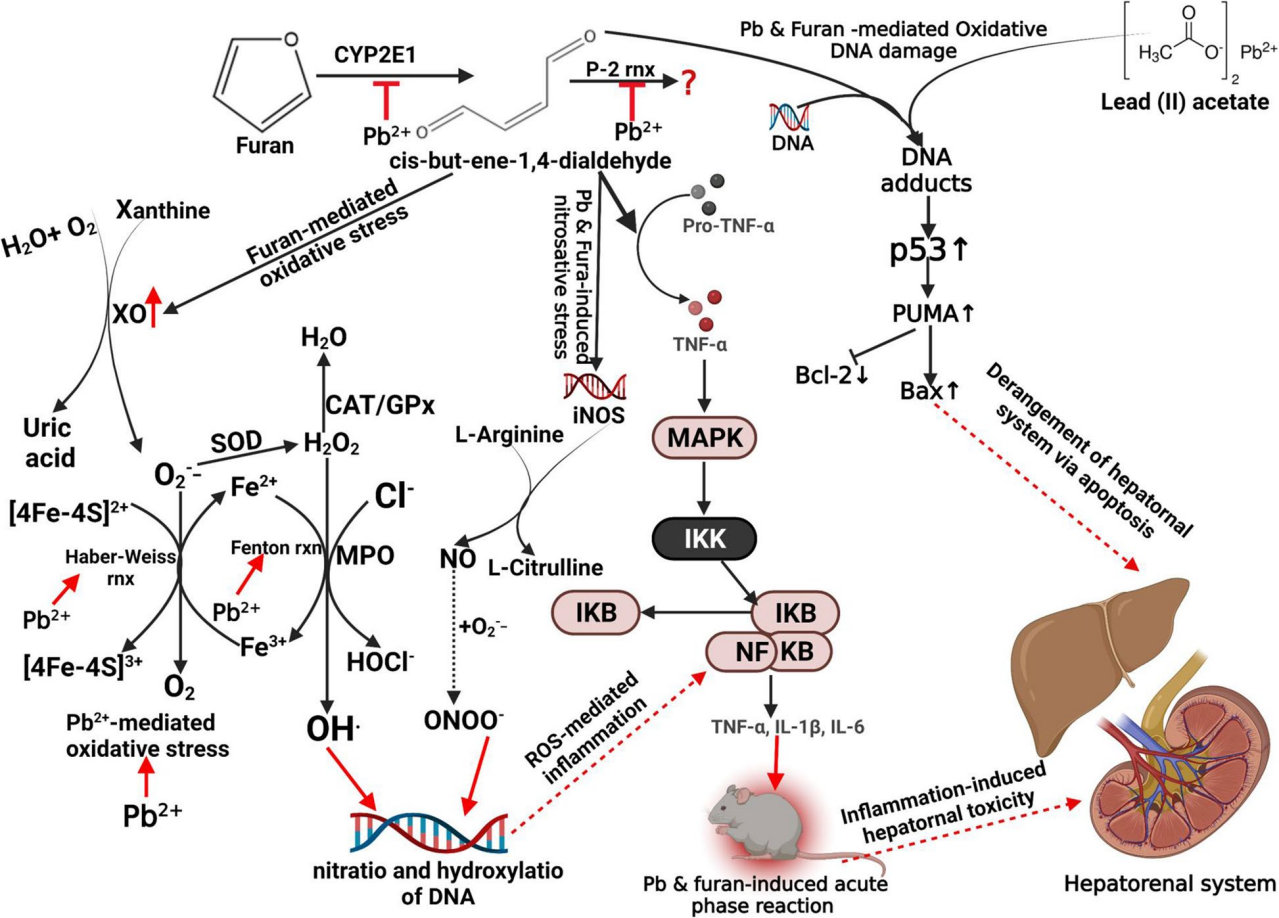
Proposed mechanisms of accidental co-exposure of experimental rats’ models to Lead and Furan in diet and water, respectively. Furan is converted to cis-but-ene-1,4-dialdehyde. This intermediate induces oxidative stress and inflammation by activating xanthine oxidase and myeloperoxidase to generate abundant ROS, including O_2_^−^, H_2_O_2_, and HOCl. On the other hand, Pb triggers the Fenton and Haber–Weiss reaction to generate abundant OH^−^. These intermediates activate iNOS which mediate the generation of NO and other pro-inflammatory cytokines such as IL-1β. Unresolved oxidative stress and inflammation will trigger oxidative DNA damage and increase the tissue level of 8-OHdG. Increases in the 8-OHdG DNA adduct will trigger the activation of p53, instructing PUMA to activate Bax while repressing the activity of Bcl-2. Bax enters the mitochondria of the hepatocytes and nephrons and causes the evolution of cytochrome C, which binds to apaf to form apoptosome with concurrent activation of caspase-9. Active caspase-9 then activates caspase-3 – the executioner of programmed cell death

The present study reveals that exposure to PbAc and furan in rats abrogated the balance in rats’ redox system, altered basal inflammatory response, triggered oxidative DNA damage, and orchestrated programmed cell death. These findings were further reinforced by lesions manifested in the forms of altered histoarchitectural features of the liver and kidney with likely impaired hepatorenal functions. Probable mechanisms for the observed biochemical changes may be through the upregulation of the activities of cytochrome P450 (CYP) 1A2, 2C19, 2C9, 2D6, and 3A4, the expression of NF-kB, p53, PUMA, Bax, caspase-9 and caspase-3, while inhibiting Bcl-2, Nrf-2, NQO1, and HO-1 (Fig. 11). Our observations recapitulate the harmful effects of the exposure to Pb and Furan on animals and warrant further molecular studies to exhaustively elucidate mechanisms of Pb and Furan toxicities in the hepatorenal system. Based on these findings, we call on different regulatory bodies in Africa to make policies against indiscriminate use of compounds containing Pb to circumvent contamination and monitor the methods for processing and preservation of food products to prevent contamination by Furan.

## Data Availability

The datasets used and analysed during the current study are available from the corresponding author upon reasonable request.

## Abbreviations

GSH: Glutathione
TSH: Total sulfhydryl group
SOD: Superoxide anion dismutase
CAT: Catalase
GPx: Gluathione peroxidase
GST: Glutathione S-transferase
XO: Xanthine oxidase
MPO: Myeloperoxidase
NO: Nitric oxide
IL-1β: Interleukin 1-beta
IL-10: Interleukin-10
Casp-9: Caspase-9
Casp-3: Caspase-3
ALT: Alanine aminotransferase
AST: Aspartate aminotransferase
ALP: Alkaline phosphatase
8-OHdG: 8-Hydroxydeoxyguanosine
GGT: Gamma glutamyl transferase
